# Effects of factor v Leiden polymorphism on the pathogenesis and outcomes of preeclampsia

**DOI:** 10.1186/s12881-019-0924-6

**Published:** 2019-11-27

**Authors:** G. K. Ababio, K. Adu-Bonsaffoh, E. Abindau, G. Narh, D. Tetteh, F. Botchway, D. Morvey, J. Neequaye, I. K. Quaye

**Affiliations:** 1 Department of Medical Biochemistry, University of Ghana School of Medicine and Dentistry, P. O. Box 4236, Accra, Ghana; 2 Department of Obstetrics and Gynecology, University of Ghana School of Medicine and Dentistry, Accra, Ghana; 3 Department of Physiology, University of Ghana School of Medicine and Dentistry, Accra, Ghana; 4 Department of Biochemistry, University of Ghana, Accra, Ghana; 5 National Diabetic Research Lab, Korle-Bu Teaching Hospital, Accra, Ghana; 6 Department of Child Health, Korle-Bu Teaching Hospital, Accra, Ghana; 7 Clinical Documentation Improvement Specialist, Southern California Hospital, Culver City, CA 90232 USA; 8 Biochemistry, Kwame Nkrumah University of Science And Technology, Kumasi, Ghana; 9 Department of Applied Health Sciences, Regent University of Science and Technology, Accra, Ghana

**Keywords:** Factor V, Leiden, Preeclampsia, Polymerase chain reaction, Restriction

## Abstract

**Background:**

Factor V Leiden polymorphism is a well-recognized genetic factor in the etiology of preeclampsia. Considering that Ghana is recording high incidence of preeclampsia, we examined if factor V Leiden is a contributory factor to its development and pregnancy outcomes.

**Methods:**

STROBE consensus checklist was adopted to recruit eighty-one (81) consenting subjects after ethical clearance. Subjects were followed up till delivery to obtain outcomes of PE. Routine blood chemistry and proteinuria were done on all samples. Factor V Leiden was characterized by polymerase chain reaction and restriction fragment length polymorphism (RFLP). The data was captured as protected health information (PHI) and analyzed with SPSS version 22.

**Results:**

Overall allelic frequencies found in FVL exon 10 were 0.67 and 0.33 for G and A alleles respectively. The FVL mutation was more in PE and hypertensive patients. Increased white blood cells, increased uric acid and a three – fold increment of AST / ALT ratio was observed in PE cases when stratified by FVL exons (exon 8 and 10). Significant differences were also observed between FVL and age, systolic blood pressure (SBP), diastolic blood pressure (DBP), liver enzymes, white blood cells (wbc), hemoglobin levels.

**Conclusion:**

FVL mutation allele frequency was 0.33, a first report. The mutation was associated with increased uric acid, liver enzymes and blood cell indices suggestive of acute inflammation.

## Background

Preeclampsia (PE) is a disease of major public health concern with high incidence of maternal and perinatal mortality [1–3]. Its etiology is multifactorial with no reliable test for predicting preeclampsia among a cohort of pregnant women. This has been complicated by the recent subclassification of preeclampsia as mild, moderate and severe or early and late onset [4]. According to the World Health Organization (WHO), maternal mortality ratio in Ghana is 350 per 100,000 live births, which is unacceptably high compared to the global ratio of 216 per 100,000 live births. Also, the maternal mortality in Korle-Bu Teaching Hospital (KBTH), which is the current study site, has been increasing since the mid-1990s from 734.4 [5] to 915.3 per 100,000 live births in 2012 [6]. This is clearly an aggravation, so it is imperative that investigations into the etiology and pathogenesis of preeclampsia is carried out in our indigenous women to help minimize its occurrence and the associated complications. We examined if mutations in Factor V Leiden (FVL) [7], contributes to the observed upward trend in Ghana. Factor V leiden, have currently been well associated with PE development. The factor V Leiden variant resists cleavage by activated protein C due to the substitution of glutamine residue with arginine at position 506 (Gln^506^), which is the cleavage site for activated protein C. The resistance is seen in inherited familial thrombophilia and venous thrombosis [8] and has recently been implicated in rampant fetal loss [9]. There is therefore anticipation that evolutionary signatures such as geographical origin, mutations, *cis* – acting elements, *trans* – acting factors and natural selection might influence genetic prediction of PE and its deadly complications. The current study sought to identify factor V leiden mutation in PE in the Ghanaian population and examine if these are associated with PE, to provide insight into a probable factor in the rise in PE, seen in the general population in pregnant women attending anti-natal care at the Korle-Bu Teaching Hospital in particular.

### Aim

To assess if factor V Leiden polymorphism contributes to preeclampsia and pregnancy outcomes in Ghanaian women.

## Method

A total of ninety-six (96) subjects were recruited by simple random sampling, consisting of thirty-two (32) each of pregnant normotensive, PE and apparently healthy non pregnant women. All patients consented in writing to participate in the study, following an explanation of the aims of the study. The study was conducted at the Korle-Bu Teaching Hospital (KBTH), Accra, Ghana. Ethical approval (**MS-Et/M.3 – P.3.2/2013–2014**) was given by the College of Health Sciences, University of Ghana Medical School Ethical Review Board.

At recruitment, each subject completed a questionnaire (Additional file 1) after which demographic and anthropometric data were obtained. Spot urine samples were collected to determine protein-creatinine ratio using a Clinitek analytic strips. Also, 24-h urine samples were collected from PE patients, starting from the time of diagnosis, to determine the level of proteinuria. Venous blood (5 ml) were taken from the antecubital vein into vacutainers. All pregnant women were observed till delivery, for outcomes of PE. For the purposes of this study, PE was defined based on American College of Obstetricians and Gynecologists criteria. Accordingly, PE was defined as diastolic blood pressure (DBP) of ≥90 mmHg and or systolic blood pressure (SBP) of ≥140 mmHg with proteinuria ≥300 mg/dl occurring after 20 weeks of gestation [3]. Early or late onset preeclampsia occurs before and at or after 34 weeks of gestation respectively [4]. The preeclamptic cases were managed strictly according to the standard management protocols for preeclampsia at the KBTH including the hospital admission, monitoring of maternal and fetal parameters, the use of antihypertensives and or magnesium sulphate and timely delivery of the baby.

### Inclusion

For PE, only patients diagnosed with PE based on the definition were recruited after obtaining their consent. Control subjects included normotensive pregnant and non-pregnant women who gave informed consent to be included in the study.

### Exclusion criteria

Patients with a history of chronic hypertension, renal disease, diabetes, urinary tract infection, cardiovascular disease, multiple pregnancy, molar pregnancy, thyroid dysfunctions and infectious diseases were excluded. Women who were less than 18 years were also excluded from the study.

### Sample processing

Following the manufacturer’s protocol for each reagent kit, Sysmex (XP 300 Diagnostics, Europe GmbH) automated analyzer was used to determine full blood count (FBC). The MindRay BS 240 (Shenzheni MindRay bio-medical Electronics Company Ltd., Shenzheni, China) automated analyzer was used to determine lactate dehydrogenase levels (LDH), liver enzymes, BUE, creatinine, lipid profiles and uric acid levels, while URS – 10A (Wellkang Ltd., Suite B 29, Harley Street, London, UK) analytic strips were used for urine analysis.

### Detection of factor V Leiden mutations

To determine factor V Leiden mutation (at exon 8 and exon 10), DNA was extracted using QIAmp mini blood kit and amplified by PCR. Following the manufacturers protocol, a total of 20 μl PCR reaction containing 0.5 units of dream Taq in green Taq buffer, 2.5 ul of extracted DNAand 250 nM of forward and reverse established primers for both exon 8 (F: CATGAGAGACATCGCCTCTG, R: GACCTAACATGTTCTAGCCAGAAG) and exon 10 (F: TGCCCAGTGCTTAACAAGACCA; R: TGT-TATCACACTGGTGCTAA). The cycling conditions for amplification of exon 8 were: After initial denaturation at 95 °C for 7 min; 35 cycles at 94 °C for 1 min, 51 °C for 1 min, and 72 °C for 1 min and followed extension by 72 °C for 10 min were likewise performed. For exon 10 the cycling conditions were: initial denaturation at 94 °C for 5 min; 36 cycles at 94 °C for 1 min, 54 °C for 1 min, and 72 °C for 1 min and followed extension by 72 °C for 10 min. For restriction analyses, 5 μl of each PCR product was digested with Mnl I restriction enzyme following manufacturers’ protocol and the digests were analyzed electropheretically with 3% agarose gel containing ethidium bromide.

### Statistical analysis

All data were entered into an excel spread sheet (Microsoft company, USA) and analyzed using SPSS version 18. Student’s *t* test was used to compare means of biochemical variables and analyses of variance (ANOVA) used to assess significance in the clinical variables. The dependent variable was FVL polymorphism. The independent variables included Body Mass Index (BMI), protein-creatinine ratio, proteinuria, full blood count (FBC), lactate dehydrogenase levels (LDH), liver enzymes, BUE (Blood urea electrolytes), creatinine, lipid profiles and uric acid levels. FVL mutations within the subject groups were assessed for conformity with Hardy-Weinberg Equilibrium by Chi square analysis.

## Results

The study was completed by eighty-one (81) subjects out of the total of 96 who were enrolled. Those who did not complete or had missing data were either ill or decided not to have their blood sample taken (Additional file 2: Table S1). The average blood pressure and weeks of maternal gestation in PE ranged from early onset (142/90 mmHg; 34 weeks) to late onset (159/96 mmHg, ≥34 weeks) respectively. The PCR product size for exon 8 and exon 10 were 150 bp and 276 bp respectively. A digest in exon 8 yielded 100 bp and 50 bp. Normal alleles in Exon 10 yielded 176 bp, 69 bp and 31 bp. Overall allelic frequencies found in FVL exon 10 were 0.67 and 0.33 for G and A alleles respectively (Table 1). There were increased FVL mutations in PE and hypertensive patients. However, the frequencies in PE and hypertensive subject were not in agreement with Hardy-Weinberg Equilibrium due to loss of heterozygosity. FVL homozygotes had lower age (*p* < 0.03), high systolic blood pressure, diastolic blood pressure (DBP), liver enzymes, white blood cells (wbc) and hemoglobin levels (Tables 2 and 3). The ratio of aspartate transferase (AST) to alanine transferase (ALT) had a 3-fold increase in PE upon stratification. Also, uric acid, white blood cell count (wbc), urine protein, systolic blood pressure, diastolic blood pressure was elevated under the same stratification in PE cases while platelet count decreased (Table 2 and 3). Post hoc analysis confirmed where differences occurred between groups (Table 4, Additional file 2: Table S2).

**Table 1:** Genotypic and allelic frequency (FV exon 10)

Genotype	ctrl	BP	PE
AA	2(0.22)^a^	5(0.20)	4(0.24)
AG	6(0.67)	3(0.12)	3(0.17)
GG	1(0.11)	17(0.68)	10(0.59)
HW chi square test	1.10	11.84	6.06
Allelic frequency in the respective groups			
G	0.44	0.74	0.68
A	0.56	0.26	0.32
Overall Allelic frequency: G = 0.67 A = 0.33			

*Ctrl* non-pregnant normotensive, *BP* pregnant normotensive, *PE* preeclampsia, *HW* Hardy–Weinberg equilibrium

^a^Number (frequency)

**Table 2:**
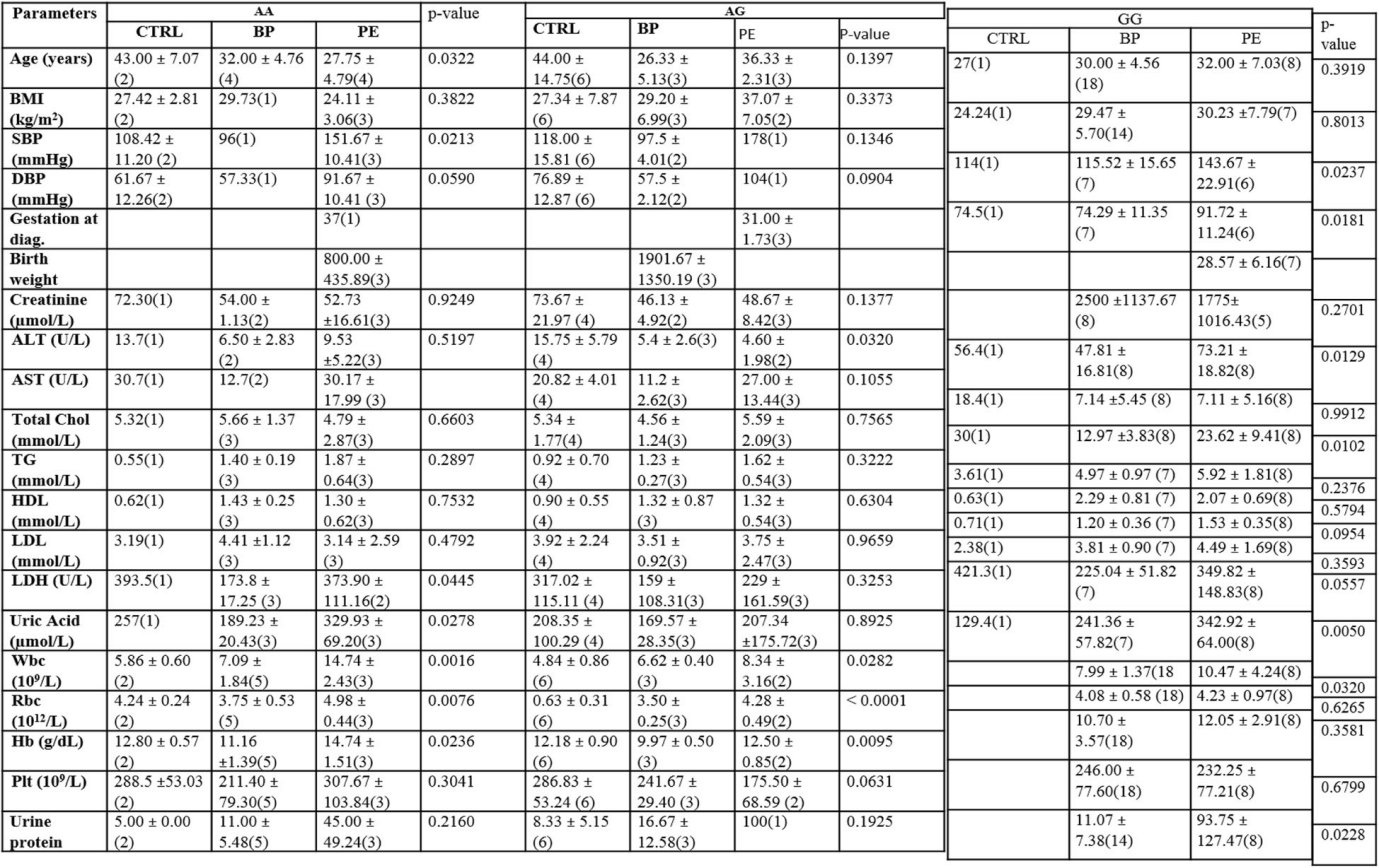
Exon 10 FVL stratification with clinical variables

Table 2 depicted three [3] genotypes stratified with clinical variables. Missing values in Ctrl was as expected since these women were apparently healthy and not pregnant. Missing data in other groups were those who were critically ill. *Ctrl* non-pregnant normotensive, *BP* pregnant normotensive, *PE* preeclampsia

**Table 3:** Exon 8 FVL stratification with clinical variables

	Exon 8 digest				
	PE	Preg. normo	Non-preg. Normo	PE x Preg. normo	PE x preg. Normo x non-preg normo
Age (years)	30.0 ± 6.3(7)	29.3 ± 5.0(12)	44.3 ± 7.8(10)	0.7920	*p* < 0.001
BMI (Kg/m^2^)	30.2 ± 8.5(6)	28.7 ± 3.5(9)	27.7 ± 5.6(10)	0.6400	0.7099
SBP (mmHg)	149.5 ± 22.6(4)	99.2 ± 4.6(4)	121.4 ± 15.8(10)	0.0048	0.0018
DBP (mmHg)	89.8 ± 17.5(4)	62.6 ± 7.1 (4)	77.3 ± 10.6(10)	0.0280	0.0176
ALT (U/L)	6.9 ± 2.7 (9)	6.1 ± 2.1 (5)	13.1 ± 4.3(7)	0.5791	0.0015
AST (U/L)	25.4 ± 9.8 (9)	12.9 ± 2.7 (5)	23.9 ± 4.7 (7)	0.0176	0.0159
Creatinine (μmol/L)	61.6 ± 14.0 (9)	48.8 ± 5.5 (5)	66.8 ± 5.9 (7)	0.0770	0.0240
Total Chol (mmol/L)	5.5 ± 1.4 (9)	4.2 ± 1.4 (5)	4.4 ± 0.7 (7)	0.1218	0.1111
TG (mmol/L)	1.6 ± 0.3 (9)	2.3 ± 0.8 (5)	0.5 ± 0.2 (7)	0.0335	*p* < 0.0001
HDL (mmol/L)	1.5 ± 0.4 (9)	0.9 ± 0.4 (5)	0.7 ± 0.3(7)	0.0197	0.0012
LDL (mmol/L)	4.2 ± 1.4 (9)	3.5 ± 1.4 (5)	2.5 ± 0.8 (7)	0.3877	0.0436
LDH (U/L)	321.7 ± 124.6 (9)	222.2 ± 50.5 (5)	317.0 ± 107.8 (7)	0.1177	0.2296
Uric acid (μmol/L)	289.1 ± 121.0 (9)	222.0 ± 50.5 (5)	165.7 ± 69.9 (7)	0.2655	0.0527
Wbc (10^9^/L)	11.5 ± 4.8 (6)	8.0 ± 1.5 (12)	5.8 ± 1.4 (11)	0.0309	0.0005
Rbc (10^12^/L)	4.4 ± 0.7 (6)	3.9 ± 0.5 (12)	4.5 ± 0.3 (11)	0.0985	0.0161
Hb (g/dL)	12.5 ± 1.7 (6)	10.3 ± 3.1 (12)	13.0 ± 0.6 (11)	0.1279	0.0167
Plt (10^9^/L)	283.5 ± 66.2 (6)	212.7 ± 46.0(12)	298.2 ± 56.8 (11)	0.0172	0.0023
Urine Protein	70.8 ± 105.9 (7)	11.4 ± 5.0 (11)	5 (0)	0.0769	
No. of preg.	3.7 ± 2.1 (6)	2.7 ± 1.3 (11)		0.2403	
No. of prev. Birth	2.2 ± 1.9 (6)	1.2 ± 0.8 (11)		0.1436	
Birth weight	1300.0 ± 1168.0 (5)	2716.7 ± 694.0 (6)		0.0337	

After multiple testing correction, age, SBP, Alt, TG, HDL, wbc and Plt remained significant. Body mass index (BMI), systolic blood pressure (SBP), diastolic blood pressure (DBP), aspartate transferase (AST), alanine transferase (ALT), total cholesterol (Total Chol), triglyceride (TG), high density lipoprotein (HDL), low density lipoprotein (LDL), lactate dehydrogenase (LDH), white blood cell (wbc), red blood cell (rbc), haemoglobin (Hb), platelet (Plt), number of pregnancies (No. of preg.), number of previous birth (No. of prev. birth)

**Table 4:** Multiple Comparisons FV exon 10

	(I) idCat	(J) idCat	Mean Difference (I-J)	Std. Error	Sig.	95% Confidence Interval	
						Lower Bound	Upper Bound
Tukey HSD	1.00	2.00	−.921^*^	.277	.010	−1.62	−.22
		3.00	−.810^*^	.306	.040	− 1.59	−.03
	2.00	1.00	.921^*^	.277	.010	.22	1.62
		3.00	.111	.290	.923	−.63	.85
	3.00	1.00	.810^*^	.306	.040	.03	1.59
		2.00	−.111	.290	.923	−.85	.63
Bonferroni	1.00	2.00	−.921^*^	.277	.011	−1.65	−.19
		3.00	−.810^*^	.306	.048	− 1.61	−.01
	2.00	1.00	.921^*^	.277	.011	.19	1.65
		3.00	.111	.290	1.000	−.65	.87
	3.00	1.00	.810^*^	.306	.048	.01	1.61
		2.00	−.111	.290	1.000	−.87	.65
Tamhane	1.00	2.00	−.921^*^	.205	.002	−1.48	−.36
		3.00	−.810	.363	.174	− 1.95	.33
	2.00	1.00	.921^*^	.205	.002	.36	1.48
		3.00	.111	.364	.988	−1.03	1.25
	3.00	1.00	.810	.363	.174	−.33	1.95
		2.00	−.111	.364	.988	−1.25	1.03

ANOVA at 95% CI, significance = 0.009, F = 6.131

Dependent variable: FV exon 10

Factor: ID catr (ID categories)

Number of samples = 57

Strata variables: age categories, BMI categories, SBP categories, DBP categories, Uric acid categories, wbc categories, LDH categories

1 = non-pregnant normotensive 2 = pregnant normotensive 3 = preeclampsia

## Discussion

In this study, a report on the effects of FVL on the outcomes of PE were presented. This study was the first to report baseline FVL genotypic and allelic frequencies as well as relating clinical variables with it. The study also reports for the first time that in the pregnant women studied, FVL mutation allele frequency being 0.33, is consistent with reports elsewhere [9]. The loss of heterozygosity in the PE and hypertensive subjects could be due to founder effects or genetic drifts [10]. These could be critically examined in a follow up study.

The strikingly three – fold increment of AST / ALT ratio, increased white blood cell (wbc) and increased uric acid levels observed in PE cases when stratified by both exons indicates that genetic disposition to FVL could be associated with susceptibility to PE and might confer poor PE outcomes. The underlying pathophysiology appears to be acute inflammation as all blood cell indices were elevated including uric acid. These were also consistent with previous findings in other populations [11–14].

Although, wbc counts [11–14], liver enzymes [11–14] and uric acid [15–19] had been suggested to increase in PE, it was notable to mention that no study associated FVL to such parameters in Ghana. More so, there had been scanty information on the impact of FVL in clinical events where inflammation was present in the country. FVL referred to a specific gene mutation which led to hypercoagulability with serious clinical consequences.

Much work has been done elsewhere on FVL [20–26] in PE; however, while others never found FVL mutation in their cohort [23], others strongly cited indications of FVL [22, 24] being a unique predictor of PE. Even though sample size in the current study was small, findings of heterozygosity and normal homozygous in FVL exon 10 seemed to be consistent with Dizon – Townson et al. [9] We could not however extrapolate the findings to the general population unless this was further interrogated with a larger sample size. We tried to minimize selection bias using two control(s) e.g. pregnant normotensive and non-pregnant normotensive per case to improve the statistical power of the study since those from the hospital setting had outcomes related to the exposure being studied.

The inflammatory response attributed to FVL might be due to platelets activating the inflammation, with uric acid channeling macrophage and dendritic cell activation, thereby increasing proinflammatory cytokines to sustain inflammation and adverse outcomes [25]. The absence of FVL mutation in exon 8 was consistent with O’Shaughnessy et al. [26] Nevertheless, the current study was able to identify mutant homozygous in FVL exon 10.

## Conclusion

FVL mutation could lead to an inflammatory state in PE. We have for the first time reported the allelic frequency in a section of the Ghanaian population.

## Supplementary information

**Additional file 1:.** Questionnaire

**Additional file 2: Table S1.** Performance profile of the Preparative Technique(s) used. **Table S2.** Bootstrap for Multiple Comparisons: Dependent Variable: FV exon 10

## Abbreviation(s)

ALT: Alanine transferase
AST: Aspartate transferase
FVL: Factor V Leiden
Gln: Glutamine
Hb: Haemoglobin
HDL: High density lipoprotein
KBTH: Korle-Bu Teaching Hospital
LDH: Lactate Dehydrogenase
LDL: Low density lipoprotein
PCR: Polymerase Chain Reaction
PE: Preeclampsia
Plt: Platelet
rbc: red blood cell
Total Chol: Total Cholesterol
Tri: Triglyceride
wbc: white blood cell
WHO: World Health Organization
