# Supplementary material for: Effects of factor v Leiden polymorphism on the pathogenesis and outcomes of preeclampsia
Source: BMC Med Genet. 2019 Nov 27;20:189. doi: 10.1186/s12881-019-0924-6 (PMC6882245; doi:10.1186/s12881-019-0924-6)
Supplement: Supplementary file 1 — Additional file 1:. Questionnaire [file 12881_2019_924_MOESM1_ESM.docx]

| **UNIVERSITY OF GHANA MEDICAL SCHOOL**  **COLLEGE OF HEALTH SCIENCES**  **TEL: 233 302 661311**  DEPARTMENT OF MEDICAL  BIOCHEMISTRY  P O Box 4236  ACCRA. GHANA |
| --- |


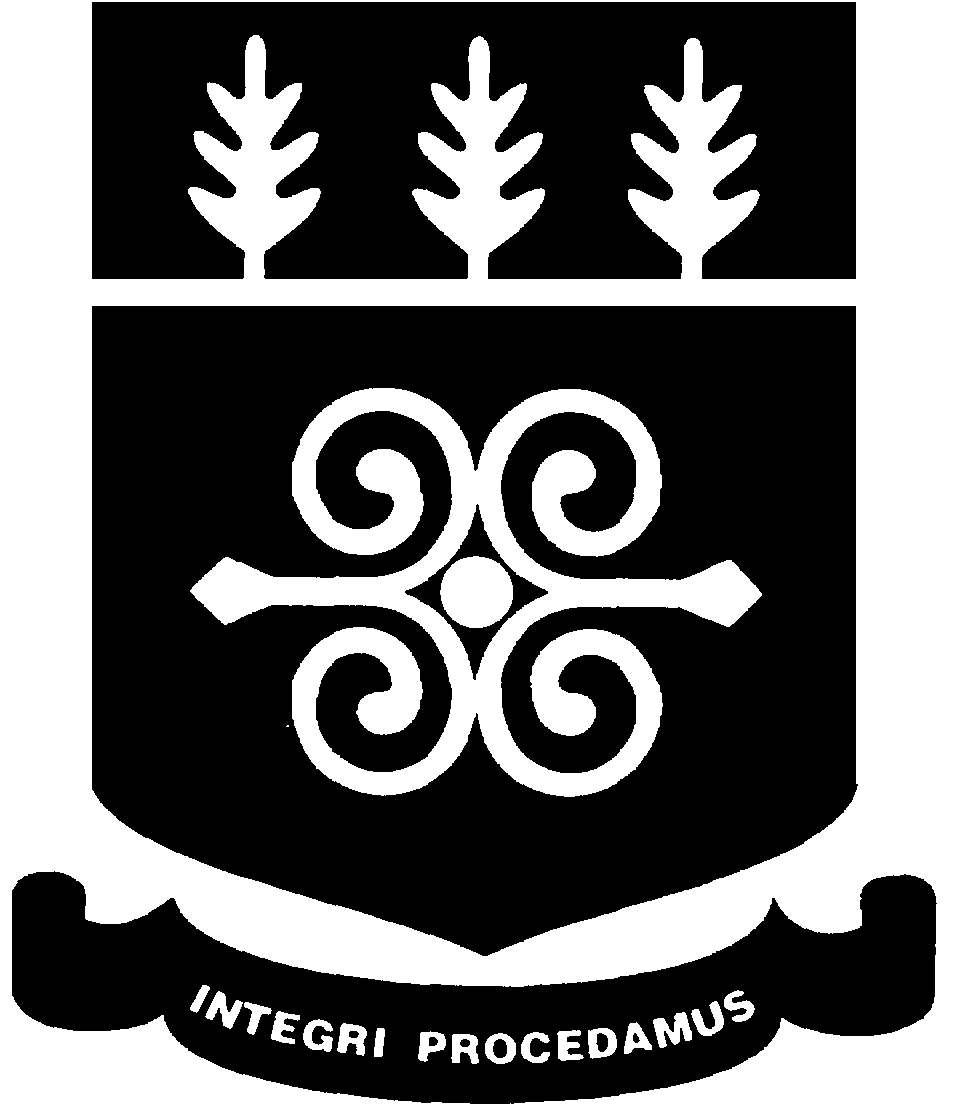


PATIENT INFORMED CONSENT FORM

Participant ID Number:

Participant Name­

Study Title: **Effects of factor V Leiden polymorphism on the pathogenesis and outcomes of preeclampsia**

You are being asked to take part in this research study because you have PE. You are kindly to understand that:

1. Taking part in the research is entirely voluntary.

2. We will take the following measurements: blood pressure, height and weight, parity, gravidity. Two tablespoons of blood will also be drawn to measure substances in blood including full blood counts, factor V Leiden and others. This amount of blood is not very different from what you will normally be asked to provide when you first come to our center.

We may find that you have early stages of PE. If that is so, we will offer you the care normally given to patients with such conditions. If you do not wish to take part in this study, it will not interfere with your care or treatment at the Obstetrics and Gynecology Centre. You may withdraw from the study at any time without anyone objecting. Do you have any questions?

CONSENT

I have fully explained to the nature and purpose of the above described study and risks that are involved in its performance. I have answered and will answer all questions to the best of my ability.

(Signature) Full Name of Staff Member Date

Dr. GK Ababio and Dr. Adu-Bonsaffoh, staffs of the University of Ghana will be available to answer any questions you may have.

If this study is reported you will not be identified by name or any other identifying information.

You have been fully informed of the above-described research study with its possible benefits and risks. Your signature or thumbprint below indicates that you are willing to participate in this research study. You do not give up any of your legal rights by signing this consent document.

(Signature/thumbprint of Subject) Date:

Date:

QUESTIONNAIRE

BACKGROUND INFORMATION:

1. Individual Identification Number………………………..
2. Date of Hospital Arrival ………………………….
3. Date of Hospital Discharge/Death
4. Duration of hospital stay………………………..
5. Occupation…………………………………
6. Marital Status:

(0: married/cohabitation 1: divorced/separated 2: Single 3: Other)

1. Which tribe do you belong to? (ethnicity)…………………….
2. Which part of the country do you live?

(0: Greater Accra, 1: Western, 2: Central, 3: Volta, 4: Eastern, 5: Others)

1. Age in years………..
2. Which religion do you belong to? (0: Christian 1: Muslim 2: Others)
3. What is the highest degree of education that you attained?

(0: None 1: Primary 2: Junior High Secondary 3: Senior High Secondary 4: Tertiary)

**Current Obstetric History**

1. Gestational age in weeks at recruitment/ diagnosis……………..
2. Number of pregnancies (including current pregnancy)…………
3. Number of previous births (excluding current delivery)…………
4. Number of antenatal visits with the current pregnancy……….……

**Maternal Outcome Indicators**

1. Height (M)………………………………
2. Weight at booking (Kg)………………....
3. BMI at booking (Kg/m^2^)………….……..
4. Gestational age at booking………………
5. Blood Pressure at Booking……………….
6. Gestational age at diagnosis……………..
7. Blood Pressure at diagnosis……………….
8. Urine protein at diagnosis………………….
9. Was the patient referred here Yes =1 No =2
10. If yes what was the reason for referring………………………
11. Where was the patient referred from?.....................................
12. Eclampsia Yes =1 No =2
13. Severe preeclampsia Yes =1 No =2
14. Haemoglobin before delivery………………………….
15. Haemoglobin after delivery……………………………

**Perinatal Outcome Indicators**

1. Sex 1. Male 2. Female
2. Birth weight (Kg)………………
3. APGAR at 1minute………….
4. APGAR at 5minutes………….

**Biochemical Investigations**

FBC ………………..

BUE creatinine………………

[Uric acid]…………………….

[AST]……………………….

[ALT]……………………….

[LDH]…………………

[Triglycerides]………………

[HDL]………………..

[LDL]………………

[cholesterol]………………..

Genetics

Factor V Leiden …………..

THANK YOU

Date of Data Collection:____________________________________

Data Collector’s Name: ____________________________________
