# Supplementary material for: Effects of factor v Leiden polymorphism on the pathogenesis and outcomes of preeclampsia
Source: BMC Med Genet. 2019 Nov 27;20:189. doi: 10.1186/s12881-019-0924-6 (PMC6882245; doi:10.1186/s12881-019-0924-6)
Supplement: Supplementary file 2 — Additional file 2: Table S1. Performance profile of the Preparative Technique(s) used. Table S2. Bootstrap for Multiple Comparisons: Dependent Variable: FV exon 10 [file 12881_2019_924_MOESM2_ESM.docx]

**Supplementary Table 1 Performance profile of the Preparative Technique(s) used**

|  | Exon 8 | Exon 10 |
| --- | --- | --- |
| Both PCR and Mnl digestion, successful | 32 | 81 |
| No show for Mnl digestion even though PCR was successful | 34 | 15 |
| Did not give consent for other clinical and biochemical investigations* | 30 | 24 |
| Successful exon stratification with clinical variables | 32 | 57 |

*Uncompromising compliance

| **Supplementary Table 2 Bootstrap for Multiple Comparisons**  Dependent Variable: FV exon 10 | | | | | | | |
| --- | --- | --- | --- | --- | --- | --- | --- |
|  | (I) idCat | (J) idCat | Mean Difference (I-J) | Bootstrap^a^ | | | |
|  |  |  |  | Bias | Std. Error | 95% Confidence Interval | |
|  |  |  |  |  |  | Lower | Upper |
| Tukey HSD | 1.00 | 2.00 | -.921 | -.007 | .161 | -1.295 | -.571 |
|  |  | 3.00 | -.810 | .012 | .146 | -1.138 | -.500 |
|  | 2.00 | 1.00 | .921 | .007 | .161 | .571 | 1.295 |
|  |  | 3.00 | .111 | .019 | .120 | -.196 | .364 |
|  | 3.00 | 1.00 | .810 | -.012 | .146 | .500 | 1.138 |
|  |  | 2.00 | -.111 | -.019 | .120 | -.364 | .196 |
| Bonferroni | 1.00 | 2.00 | -.921 | -.007 | .161 | -1.295 | -.571 |
|  |  | 3.00 | -.810 | .012 | .146 | -1.138 | -.500 |
|  | 2.00 | 1.00 | .921 | .007 | .161 | .571 | 1.295 |
|  |  | 3.00 | .111 | .019 | .120 | -.196 | .364 |
|  | 3.00 | 1.00 | .810 | -.012 | .146 | .500 | 1.138 |
|  |  | 2.00 | -.111 | -.019 | .120 | -.364 | .196 |
| Tamhane | 1.00 | 2.00 | -.921 | -.007 | .161 | -1.295 | -.571 |
|  |  | 3.00 | -.810 | .012 | .146 | -1.138 | -.500 |
|  | 2.00 | 1.00 | .921 | .007 | .161 | .571 | 1.295 |
|  |  | 3.00 | .111 | .019 | .120 | -.196 | .364 |
|  | 3.00 | 1.00 | .810 | -.012 | .146 | .500 | 1.138 |
|  |  | 2.00 | -.111 | -.019 | .120 | -.364 | .196 |
| a. Unless otherwise noted, bootstrap results are based on 57 stratified bootstrap samples | | | | | | | |

Strata variables: age categories, BMI categories, SBP categories, DBP categories, Uric acid categories, wbc categories, LDH categories

1 = non-pregnant normotensive 2 = pregnant normotensive 3 = preeclampsia
